# Sulfonated (1→6)-β-d-Glucan (Lasiodiplodan): Preparation, Characterization and Bioactive Properties

**DOI:** 10.17113/ftb.57.04.19.6264

**Published:** 2019-12

**Authors:** Gabrielle Cristina Calegari, Vidiany Aparecida Queiroz Santos, Aneli M. Barbosa-Dekker, Cleverson Busso, Robert F. H. Dekker, Mário Antônio Alves da Cunha

**Affiliations:** 1 Chemistry Department, Federal University of Technology - Paraná, Via do Conhecimento, Km 1, 85503-390 Pato Branco, PR, Brazil; 2 Chemistry Department, State University of Londrina, Rod. Celso Garcia Cid, Km 380, 86057-970 Londrina, PR, Brazil; 3 Bioprocess and Biotechnology Engineering Coordination, Federal University of Technology - Paraná, Rua Cristo Rei, 19, 85902-490 Toledo, PR, Brazil; 4 Graduate Program in Environmental Engineering, Federal University of Technology - Paraná, Estr. dos Pioneiros, 3131, 86036-370 Londrina, PR, Brazil

**Keywords:** sulfonation, fungal exopolysaccharides, microbiocidal activity

## Abstract

Sulfonated derivatives of lasiodiplodan (LAS-S) with different degrees of substitution (1.61, 1.42, 1.02 and 0.15) were obtained and characterized by Fourier-transform infrared spectroscopy (FTIR), scanning electron microscopy (SEM), and thermal and solubility analyses. Antimicrobial, antioxidant and cytotoxic potential were also assessed. The sulfonation was confirmed by FTIR analysis with specific bands at 1250 cm^-1^ (S=O, strong asymmetrical stretching vibration) and at 810 cm^-1^ (C-O-S, symmetrical vibration associated with the C-O-SO_3_ group) in the sulfonated samples. SEM demonstrated that sulfonation promoted morphological changes on the surface of the biopolymer with heterogeneous fibrillary structures appearing along the surface following chemical modification. LAS-S showed high thermal stability, with mass loss due to oxidation at temperatures close to 460 °C. Sulfonation increased the solubility of LAS, and in addition, increased the antimicrobial activity, especially against *Candida albicans* (fungicidal) and *Salmonella enterica* Typhimurium (bacteriostatic). Native lasiodiplodan (LAS-N) showed higher OH˙ removal capacity, while LAS-S had higher ferric ion reducing antioxidant power (FRAP) potential. LAS-N and LAS-S did not demonstrate lethal cytotoxicity against wild and mutant strains of *Saccharomyces cerevisiae*. Samples with higher degree of substitution (1.42 and 1.61) showed lower potential to induce oxidative stress.

## INTRODUCTION

Polysaccharides from bacteria, yeasts, filamentous fungi and algae have been described in the scientific literature as materials with different biological potentialities. Many of these biomacromolecules have attracted attention from the pharmaceutical industry because they have immunomodulatory, antioxidant, anti-inflammatory, antimicrobial and antitumour activities, and some contribute to reduction of the risks leading to the development of cardiovascular diseases and diabetes (*1*–*3*).

Among the microbial polysaccharides, the β-d-glucans, biopolymers composed of glucose units linked by glucosidic bonds in the β-anomeric configuration, stand out with their innumerable possibilities of applications in the most varied areas. The biological and technological functions of β-glucans are directly associated with the macromolecular structure of these biomolecules. Size of the polymer chains, spatial conformation and branching types are relevant parameters that strongly influence their properties (*1*). In this context, the search for new glucans, including the chemically modified derivatives, which may present new biotechnological functions or have their original properties enhanced, is a strategic and promising tool.

Sulfonation is highlighted among the different chemical modifications of polysaccharides that can present novel biological functions. It is a reaction that involves the nucleophilic substitution of the hydroxyl groups from the monomeric sugar units by sulfonyl hydroxide group (S(=O)_2_(OH)). Derivatization reactions can change the properties of polysaccharides, and in the case of sulfonation present new functions such as anticoagulation, antithrombotic, antiviral and antimicrobial activities (*4*, *5*).

Chemically modified polysaccharides have increasingly attracted the attention of researchers and industry for their interesting technological properties, and physiological and biological functions. These biopolymers today constitute a niche market involving millions of dollars, with prospects of expansion especially in the pharmaceutical and food sectors. The global biopolymer market was valued at US$ 2.5 million in 2017, and is estimated to grow at annual growth rate of 15.2%, reaching an estimated value of US$ 7.2 million by 2024 (*6*).

Based on the aforementioned information, and building on earlier studies on the fungal carbohydrate biopolymer, lasiodiplodan, that was developed in our laboratory (*7*), the present study focuses on the fermentative production of lasiodiplodan and its chemical derivatization by sulfonylation. Lasiodiplodan is a fungal exocellular linear d-glucan of the type (1→6)-β-d-glucan produced by the ascomycete *Lasiodiplodia theobromae* MMPI that has demonstrated different biological functions, including antioxidant, hypocholesterolemic, hypoglycemic and anticarcinogenic activities (*2*).

Lasiodiplodan-derivatized macromolecules with different degrees of substitution were obtained, characterized chemically, and evaluated for potential biological activities, such as antimicrobial and antioxidant activity. In addition, the oxidative stress potential of the derivatives on recombinant strains of *Saccharomyces cerevisiae* was also evaluated. As an innovative aspect in obtaining sulfonated lasiodiplodan, we report on the use of a new sulfonation protocol, where the sulfonating agent (chlorosulfonic acid) and catalyst (pyridine) were first mixed and then added directly to the solubilized polysaccharide. Different relationships between the concentrations of derivatizing agent and catalyst, and their effects on the degree of substitution of the derivatives are also evaluated.

## MATERIALS AND METHODS

### Microorganism and chemicals

The β-glucan (lasiodiplodan) studied in this work was produced by the ascomyceteous fungus, *Lasiodiplodia theobromae* MMPI, and maintained in the culture collection of the Bioprocess and Food Technology Research Group of the Federal University of Technology, Paraná, Brazil. The fungus was kept on Sabouraud agar medium containing chloramphenicol (antibiotic), and was subcultured at four-month intervals.

All chemicals used in the sulfonation protocols and in the determination of the degree of substitution, solubility, antioxidant and antimicrobial activities were purchased from Sigma-Aldrich Company (Merck, St. Louis, MO, USA). Glucose and mineral salts used in the preparation of fermentation media, and Sabouraud agar culture medium were purchased from Synth Company (Sao Paulo, SP, Brazil).

### Lasiodiplodan production

Lasiodiplodan was produced by submerged fermentation in 250-mL Erlenmeyer flasks on nutrient medium (135 mL) comprising minimal salt medium (MSM) (*8*) and glucose (20 g/mL), and was inoculated with a standardized fungal inoculum (15 mL) as described by Alves da Cunha *et al*. (*9*). The flasks were kept in a shaker incubator (TE 4200; Tecnal, Piracicaba, Brazil) at 28 °C for 72 h and 150 rpm agitation. At the end of the process, the fermented broth was separated from the mycelial biomass by centrifugation (1500×*g* for 15 min) in a digital bench centrifuge (NT 810; Novatecnica, Piracicaba, SP, Brazil). The exopolysaccharide was precipitated from the fermentation broth with 95% *(V*/*V*) ethanol at 5 °C for 12 h, and the precipitate was collected by filtration, resolubilized in water, and exhaustively dialyzed for 5 days against distilled water (MMCO dialysis tubes 12 000 Da, 1.3 in; Sigma-Aldrich, Merck), and then lyophilized. Under the conditions of fermentation and recovery of the biopolymer, a high purity polysaccharide (97% total carbohydrate (as glucose) and 2-3% protein) was obtained.

### Lasiodiplodan sulfonation

The sulfonation reagent was prepared according to Lu *et al*. (*10*) with modification. To obtain sulfonated derivatives of lasiodiplodan with different degrees of substitution (DS), four ratios of chlorosulfonic acid (CSA) to pyridine (Py) of 1:4, 1:5, 1:6 and 1:10 were examined. The CSA/Py ratios were chosen to assess both, a low (1:4) and a high (1:10) catalyst (Py) amount in the reaction with derivatizing agent (CSA). The sulfonating reagent was prepared in two-necked flasks (kept in an ice bath) by allowing chlorosulfonic acid to drop onto pyridine for 40 min under constant stirring.

Derivatization of lasiodiplodan was performed following the procedure described by Zhang *et al*. (*11*) with subtle adaptation. In a two-necked flask, 200 mg of LAS-N were solubilized in 20 mL of concentrated formamide under vigorous stirring for 72 h at room temperature. Then, sulfonated reagent (20 mL) as prepared above was added dropwise, and the mixture was stirred for 3 h at 60 °C. The sulfonation reaction was terminated using NaOH solution (15% *m*/*V*) until the pH was neutral. Next, the resulting solution was extensively dialyzed against distilled water for 6 days and the dialysate was lyophilized and denoted as LAS-S.

### Chemical characterization

#### Determination of the degree of substitution

The degree of substitution (DS) was determined based on the correlation between the sample sulfur and carbon contents (*12*). It is defined as the average number of sulfonyl hydroxide groups (S(=O)_2_-(OH)) inserted onto each monosaccharide (glucose) unit. The sulfur and carbon contents present in LAS-S samples were determined on a CHNS/O 2400 series II Elemental Analyzer (Perkin Elmer, Waltham, MA, USA), and the DS was estimated using the following equation:
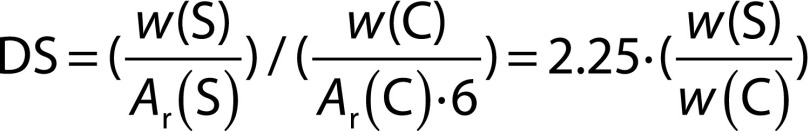
where *w*(S) and *w*(C) are mass fractions of sulfur and carbon in the sample, respectively, *A*_r_(S) and *A*_r_(C) are the atomic masses of sulfur and carbon, respectively, 6 is the number of carbon atoms per monomeric unit, and 2.25 is the ratio between *A*_r_(C)·6 and *A*_r_(S). The analyses were performed in triplicate and the results are expressed as the average values.

#### Evaluation of solubility of lasiodiplodan

Solubility of lasiodiplodan in water and 20% (*V*/*V*) DMSO was evaluated according to Wang *et al.* (*13*) with adaptations. LAS-N and LAS-S samples (10 mg) were suspended in distilled water (10 mL) and stirred for 24 h at 25 °C. The resulting solution was centrifuged at 1500×*g* (NT 810; Novatecnica) for 10 min, then the supernatant was collected and used for quantification of total sugars by phenol-sulfuric acid method (*14*), which is directly related to the soluble sample content. The solubility in water was expressed as mass of soluble polysaccharide in sample (%). Solubility in 20% DMSO was also evaluated following a similar protocol, but replacing water with 20% DMSO solution (*15*).

#### Fourier transform infrared spectroscopy analysis

Fourier transform infrared spectroscopy (FTIR) spectra of LAS-N and LAS-S were obtained on a Frontier spectrometer (Perkin Elmer, Waltham, MA, USA) in the spectral range of 4000-400 cm^-1^, with a resolution of 2 cm^-1^ and 16 scans accumulated for each spectrum, using the KBr disk technique.

#### Scanning electron microscopy

Micrographs of the lyophilized LAS-N and LAS-S samples were obtained using a scanning electron microscope TM3000 (Hitachi, New York, NY, USA). Images with magnitudes of 300, 800 and 1200× were acquired from samples attached to a carbon tape.

#### Thermal analysis

Lyophilized LAS-N and LAS-S samples were submitted to derivative thermogravimetry (DTG), differential thermal analysis (DTA) and thermogravimetric analysis (TGA), performed on an SDT (simultaneous DSC/TGA) Q600 instrument (TA Instruments, New Castle, DE, USA). The mass loss was monitored between 25 and 800 °C, at a heating rate of 10 °C/min, in an atmosphere of synthetic air with a flow rate of 50 mL/min.

### Biological characterization of native and sulfonated lasiodiplodan

#### Evaluation of antimicrobial activity

The potential antimicrobial activity was determined according to Krichen *et al.* (*16*) with modifications, on four bacterial strains (*Escherichia coli* ATCC 25922, *Listeria monocytogenes* ATCC 19111, *Staphylococcus aureus* ATCC 25923 and *Salmonella enterica* Typhimurium ATCC 0028) and two yeast strains (*Candida albicans* ATCC 10231 and *Candida tropicalis* ATCC 13803).

Antimicrobial assays were conducted in 96-well ELISA plates. Into each well 100 μL of samples (LAS-N and LAS-S separately at different concentrations: 0.26, 0.20, 0.15, 0.10 and 0.05 mg/mL), 100 µL of Mueller-Hinton broth (Sigma-Aldrich, Merck) for bacteria or Sabouraud with chloramphenicol agar (Synth) for yeast, and 5 µL of previously standardized microbial suspension in McFarland turbidity standard 0.5 (1.5·10^8^ CFU/mL) were added. The ELISA plates were incubated for 24 h at 28 °C (yeasts), or 37 °C (bacteria), and then 20 µL of resazurin dye (0.01%; Sigma-Aldrich, Merck) were added to each well to evaluate the presence of viable cells. The plates were read after 2 h and viable cells were indicated by the appearance of pink colour, while blue staining indicated cell inhibition. Positive samples for determining microbial inhibition were plated on Petri dishes containing brain heart infusion agar (bacteria) or Sabouraud with chloramphenicol agar (yeasts), and incubated in a bacteriological oven (NT 522; Novatecnica) set at 28 °C (yeast) or 37 °C (bacteria) for 24 h to verify possible bactericidal or fungicidal activity of the samples. The antimicrobials tetracycline (bacteria) and fluconazole (yeast) were used as positive controls, while sterilized peptone water (0.1%) was used as a negative control.

#### Evaluation of antioxidant activity

Ferric reducing antioxidant power (FRAP) was evaluated based on a protocol described by Wootton-Beard *et al*. (*17*). In this test, 90 μL of the lasiodiplodan samples at different concentrations (0.10, 0.18 and 0.26 mg/mL) were mixed with distilled water (270 μL) and FRAP reagent (2.7 mL). The mixture was stored in the dark for 30 min at 37 °C, and the absorbance was read in a UV/Vis spectrophotometer (Hitachi U-2800; Lambda Advanced Technology, Wembley, UK) at 595 nm. FRAP reagent was used as a blank. Results were expressed in mmol/L Fe(II) from a calibration curve of iron(II) sulfate (0.2-2.0 mmol/L).

The hydroxyl radical scavenging potential was evaluated based on that described by Liu *et al*. (*18*). Mixtures (2 mL) containing 0.5 mL FeSO_4_ (1.5 mmol/L), 0.35 mL H_2_O_2_ (6 mmol/L), 0.15 mL sodium salicylate (20 mmol/L) and 1 mL lasiodiplodan sample at different concentrations (0.10, 0.18 and 0.26 mg/mL) were incubated in tubes for 1 h at 37 °C. Thereafter, the absorbance was measured at 562 nm. Ascorbic acid (1 mg/mL) was used as a positive control. The percentage of sequestration of OH˙ was determined according to the following equation:
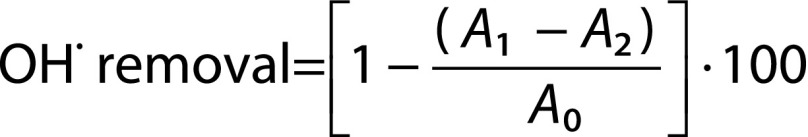
where *A*_0_ is the absorbance of the control, *A*_1_ is the absorbance of the sample or ascorbic acid, and *A*_2_ is the absorbance of the blank with only sodium salicylate.

The 2,2-diphenyl-1-picrylhydrazyl (DPPH) radical-scavenging activity was analyzed following the method described by Locatelli *et al*. (*19*) with adaptations. Samples of 0.50 mL of different lasiodiplodan concentrations (0.10, 0.18 and 0.26 mg/mL), 3 mL absolute ethanol, and 0.30 mL ethanolic DPPH solution (0.5 mmol/L) were mixed in test tubes. After 80 min of reaction, the absorbances were read at 517 nm. Blank contained only ethanol solution in water (80%), while the control contained all of the ingredients of the reaction mixture, with the ethanol solution replacing the lasiodiplodan samples. The percentage of DPPH radical scavenging activity was estimated according to the following equation:
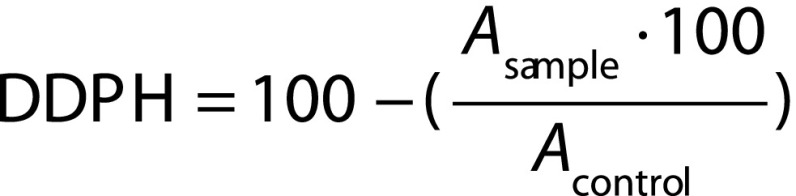
where *A*_sample_ and *A*_control_ are the absorbances of the sample control, respectively.

The 2,2′-azino-bis (3-ethylbenzthiazoline-6-sulfonic acid) (ABTS) radical scavenging activity was determined according to Wootton-Beard *et al*. (*17*) with adaptations. ABTS cation radical (ABTS^+^˙) was obtained by mixing 5 mL ABTS solution (7 mmol/L) with 88 μL potassium persulfate solution (140 mmol/L) at room temperature for 16 h. Then, the ABTS^+^˙ solution (1 mL) was diluted in absolute ethanol to an absorbance of 0.70 at 734 nm. In test tubes, lasiodiplodan samples (30 μL) at different concentrations (0.10, 0.18 and 0.26 mg/mL) were mixed with ABTS^+^˙ solution (3 mL) and shaken vigorously in the dark for 6 min, and then the absorbance was measured at 734 nm. DMSO (20% in water) was used as a blank reagent. The results are expressed in mmol/L of (±)-6-hydroxy-2,5,7,8-tetramethylchromane-2-carboxylic acid (Trolox) equivalent per mL as determined from a standard Trolox curve (0.1, 0.25, 0.5, 1.0, 1.5 and 2.0 mmol/L).

#### Evaluation of cytotoxicity on *Saccharomyces cerevisiae* cells

The cytotoxic activity of LAS-S and LAS-N was evaluated by measuring the oxidative stress capacity of wild and mutant strains of *Saccharomyces cerevisiae* as biological models. *Saccharomyces cerevisiae* ex r.f. *bayanus* (Fermol Perlage, AEB Bioquimica Latino Americana, São José dos Pinhais, Brazil) and *S. cerevisiae* mutant strains (YLL060C, GH1YIR038C and YSL101C) were evaluated.

The *S. cerevisiae* strains YLL060C, GH1YIR038C and YSL101C are mutants whose genes *GTT1* (glutathione transferase 1), *GTT2* (glutathione transferase 2) and *GSH1* (glutamate cysteine ligase) were respectively disrupted by the *KanMX4* gene (Euroscarf, Frankfurt, Germany), so they are deficient in the enzyme glutathione (GSH and GSSG). The yeast strains were maintained on yeast extract, peptone, dextrose (YPD) broth (Sigma-Aldrich, Merck) at 5 °C.

The potential to induce oxidative stress in the LAS-N and LAS-S samples was evaluated based on the protocols described by Castro *et al*. (*20*) and Subhaswaraj *et al*. (*21*) with adaptations. Assays were conducted in 96-well ELISA plates. Into each well LAS-S and LAS-N samples (100 μL separately at concentrations 0.10, 0.20 or 0.26 mg/mL), 100 μL YPD broth and 20 μL of the microbial suspension standardized on the 0.5 McFarland scale (1.5·10^8^ CFU/mL) were added. The plates were incubated at 28 °C for 24 h, and then 20 μL resazurin dye (0.01% in water) were added to each well and the plates were incubated for a further 2 h to verify the presence (pink staining) or absence (bluish staining) of viable cells. The wells with bluish staining (microbial inhibition) were inoculated onto agar plates containing YPD agar, and incubated at 28 °C for 24 h to demonstrate the absence of cell viability (cell death), or only inhibitory effect on the evaluated yeasts (colonies grown on the YPD plates). Menadione (Sigma-Aldrich, Merck) was used as a positive control, and peptone water 0.1% as negative control.

### Statistical analysis

Analytical results of antioxidant activity and solubility were expressed as the mean value of triplicate analyses. The mean values were compared by Tukey's test (p<0.05). The homogeneity of variance was checked by Levene's test, and normal distribution of results was checked using the Shapiro-Wilk test at the 5% significance level using Statistica 8.0 software (*22*).

## RESULTS AND DISCUSSION

### Degree of sulfonation

**Fig. 1:**
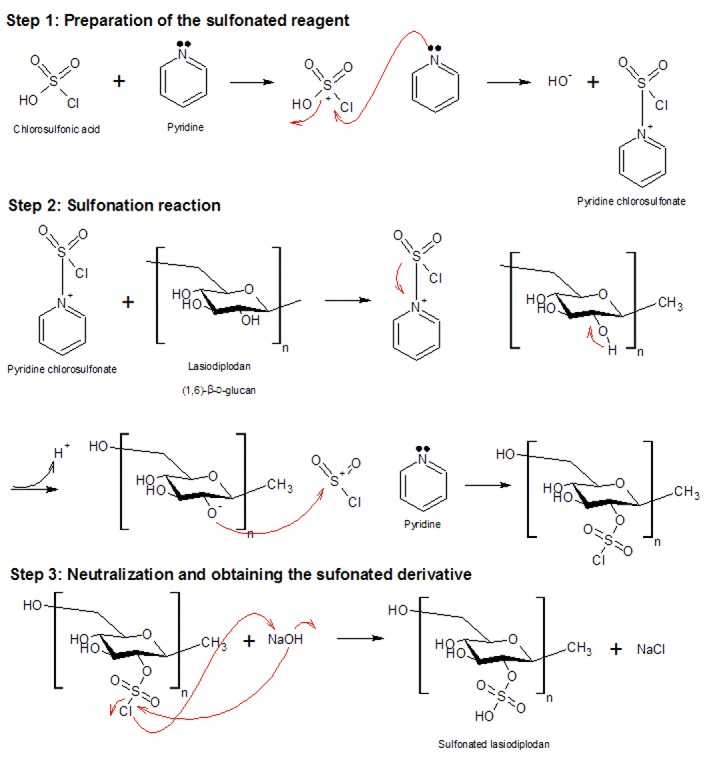
Reaction mechanism of the *O*-sulfonation of lasiodiplodan

The sulfonation reaction of native lasiodiplodan (LAS-N) can be divided into three steps (Fig. 1). The first one consists in preparing the sulfonating reagent by using a mixture of chlorosulfonic acid (sulfonic group donor) and pyridine (catalyst). In the second step, the reaction proceeds between the pyridine chlorosulfonate salt and the polysaccharide sample. In this case, the hydrogen of one hydroxyl group from LAS-N is removed and the sulfonyl group from the previously formed pyridine chlorosulfonate salt is inserted in the macromolecule. In parallel, the pyridine catalyst is released into the reaction medium. In the third and final step, the reaction mixture is neutralized with NaOH, which forms sodium chloride, and an alkali hydroxyl group binds to the macromolecule generating the stable sulfonated product (Fig. 1).

The use of different ratios of chlorosulfonic acid and pyridine (CSA/Py=1:4, 1:5, 1:6 and 1:10) in the derivatization leads to the production of sulfonated derivatives with different degrees of sulfonation (DS=1.42, 1.61, 1.02 and 0.15). When ratios 1:4 and 1:5 were used, derivatives with higher DS values (1.42 and 1.61, respectively) were obtained, and there appears to be a correlation between the DS and the CSA/Py ratio. The ratio of derivatizing agent (CSA) to catalyst (Py), temperature and time of the reaction were the main factors affecting the efficacy of derivatization by this sulfonylation protocol (*10*, *23*).

The use of higher CSA volume fractions contributed to higher DS. However, when comparing the sulfonated lasiodiplodan (LAS-S) samples obtained using the ratios CSA-/Py 1:4 (LAS-S 1:4, 25% *V*/*V* CSA) and CSA/Py 1:5 (LAS-S 1:5, 20% *V*/*V* CSA), a small difference was found among the DS values. Under such conditions of derivatization, a higher DS value was found when using 20% *V*/*V* CSA (LAS-S 1: 4) than when using 25% CSA (LAS-S 1:5). This effect may possibly be explained by the low solubility of the pyridine chlorosulfonic salt, which is obtained at higher volume fractions of CSA used in the derivatization process. In this context, preliminary tests showed that the use of volume fraction of 28% CSA in the derivatizing mixture (CSA/Py) made sulfonation unfeasible, since the generated pyridine chlorosulfonic salt began to crystallize.

Alternatively, the use of lower CSA/Py ratios (1:6 and 1:10) led to the production of derivatives with lower DS values (1.02 and 0.15, respectively). These results suggest that to obtain derivatives with higher sulfonation, it is necessary to use higher volume fractions of chlorosulfonic acid. It is noteworthy, however, that the combined use of chlorosulfonic acid and pyridine appears to be more effective in sulfonation than adding chlorosulfonic acid dropwise onto the polysaccharide and pyridine mixture. This finding is based on comparing the data from the present study with previous work (*7*).

In the present study derivatives with higher degrees of sulfonation were obtained by comparison to our previous work (DS 0.24) (*7*), although, here, the chlorosulfonic acid amounts used in the sulfonation reaction were proportionally lower (chlorosulfonic acid/lasiodiplodan ratio). Callegari *et al*. (*7*) used a ratio of 4 mL of chlorosulfonic acid to 50 mg LAS-N, and allowed the sulfonation reaction to proceed for 17 h at room temperature. In the present study, a lower ratio of chlorosulfonic acid (1.8 to 4 mL) and LAS-N (200 mg) was used, and sulfonation was conducted at a higher temperature (60 °C) for a shorter reaction time (3 h).

The derivatization conditions greatly interfered with the degree of substitution of the obtained LAS-S. Another important aspect to be highlighted is the way sulfonation is conducted. In the previous study (*7*), the polysaccharide (LAS-N) was solubilized in DMSO and initially mixed with the pyridine catalyst, followed by the addition of chlorosulfonic acid to the mixture. In the present work, LAS-N was solubilized in formamide, which was then added dropwise to a previously prepared mixture of chlorosulfonic acid and pyridine. The derivatization conditions employed in this study proved to be more effective than the conditions initially studied and reported by our group (*7*).

Assuming that it is possible to obtain a maximum DS=3 on each glucose unit in LAS-N, which has three potentially free hydroxyl groups (C-2, C-3 and C-4) for substitution, the efficiency of the sulfonation reaction of LAS-N can be estimated as reported below. The estimated efficiencies of sulfonation were: 47.33% (LAS-S 1:4 with DS=1.42), 53.67% (LAS-S 1:5 with DS=1.61), 34% (LAS-S 1:6 with DS=1.02) and 5% (LAS-S 1:10 with DS=0.15).

Sulfonated polysaccharides with DS greater than 0.8 have been highlighted in the scientific literature as bioactive macromolecules (*24*-*26*). In this sense, it is important to develop efficient sulfonation protocols that promote the derivatization of polysaccharides to obtain higher DS values.

### Solubility of the obtained derivatives

Structural modifications of polymers can promote various changes in their chemical, physicochemical and biological properties (*5*). The insertion of heteroatoms or chemical groups in the polysaccharide structure can lead to changes in interactions with water or other solvents, which may increase or decrease their solubility (*2*). In this study, the solubility of LAS-N and LAS-S samples in water and aqueous DMSO (20%) at room temperature was evaluated, and the results are shown in Table 1.

**Table 1:** Solubility of native (LAS-N) and sulfonated lasiodiplodan (LAS-S) samples in water and 20% DMSO

Sample	DS	Solubility/%	
Water	DMSO (20%)
LAS-N	-	6.14±0.00	8.720±0.007
LAS-S 1:10	0.15	7.550±0.002	11.70±0.02
LAS-S 1:6	1.02	11.590±0.002	17.050±0.008
LAS-S 1:4	1.42	8.030±0.003	10.420±0.005
LAS-S 1:5	1.61	15.350±0.007	26.500±0.005

The results are expressed as the mean value±standard deviation. LAS-S samples were produced using different chlorosulfonic acid/pyridine ratios. DS=degree of substitution

LAS-N has low solubility in water, even though it contains polar clusters such as hydroxyl groups. This can be explained by the linear structure and conformational state of the triple helix of the molecule (*25*) associated with the probable occurrence of several intramolecular interactions with the hydroxyl groups leading to the formation of macromolecular aggregates, which hinder its solvation.

As shown in Table 1, the LAS-N sample showed a solubility of 6.14% in water, and 8.720% in DMSO (20%). Similarly, all sulfonated samples showed solubility in 20% DMSO, which was somewhat higher than in water. The sulfonation reaction contributed to increased solubility in both water and 20% DMSO of the LAS-S samples, and increased solubility occurred with increasing DS.

The sample LAS-S 1:10 (DS=0.15) showed solubilities of 7.550 and 11.70% in water and 20% DMSO, respectively. When the lasiodiplodan DS was raised to 1.61, solubilities of 15.350% in water and 26.500% in 20% DMSO were found.

The introduction of large and polar chemical groups into polysaccharide macromolecules may contribute to increased solubility in water. The presence of a greater number of sulfonyl hydroxide groups can contribute to the distance of the hydroxyl groups from the macromolecules due to their large volume. The presence of a greater number of sulfonyl hydroxide group (S(=O)_2_-(OH)), and a larger intramolecular distance may lead to the appearance of a polar layer, which favours the interaction of hydrogen with water, and consequently increases solvation. On the other hand, lower solubility of the sulfonated derivatives with low DS than of derivatives with higher DS can be explained by interactions occurring between the oxygen from the sulfonyl hydroxide group and hydrogens on the unsubstituted hydroxyls of the glucose ring in the polysaccharide molecule.

It is important to note that water and DMSO solubility of the derivatives with DS values of 1.02 and 1.42 did not follow a linear correlation. Such behaviour could be explained by the relatively small differences between the substitution of the two derivatives. Another aspect that needs to be considered is that solubility is also related to the spatial arrangement of the macromolecule and the consequent exposure of hydroxyl and sulfonyl hydroxide groups.

### FTIR spectra of LAS-N and LAS-S

**Fig. 2:**
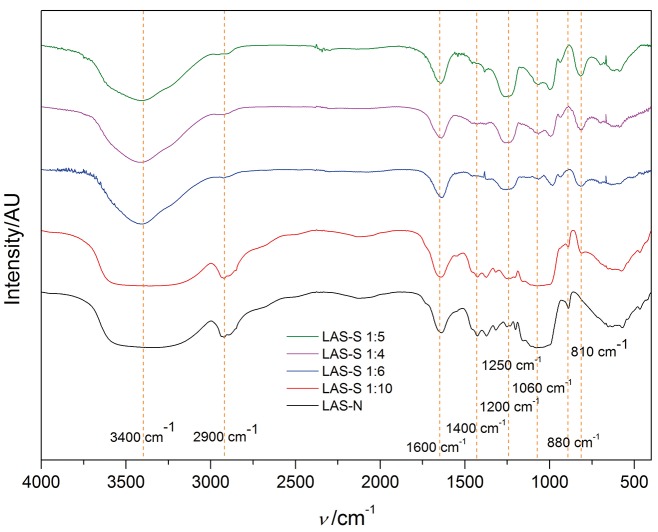
FTIR spectra of native lasiodiplodan (LAS-N) and the sulfonated derivatives with different chlorosulfonic acid/pyridine ratios (CAS/Py) that contributed to the different degrees of substitution (DS) of sulfonated lasiodiplodan LAS-S. CAS/Py=1:10 (DS=0.15),1:6 (DS=1.02), 1:4 (DS=1.42) and 1:5 (DS=1.61)

Fig. 2 shows the FTIR spectra of LAS-N and LAS-S. Typical polysaccharide signals with bands characteristic of glucans were verified. The large band of strong intensity in the 3400 cm^-1^ region is attributed to O-H stretch vibration (*4*, *16*) as free hydroxyls present in LAS-N and LAS-S. The band at approx. 2900 cm^-1^ corresponds to stretch C-H sp_3_ (*4*), present in polysaccharides such as the β-d-glucans, which have many carbon-hydrogen connections. The band of medium intensity at 1600  cm^-1^ was attributed to the stretching vibration of the glucose ring (*27*). Low-intensity bands between 1400 and 1200 cm^-1^ indicate C-H and O-H deformation vibrations typical of carbohydrates (*7*), which may have small variations in intensity and wavenumber depending on the nature of the polysaccharide (*28*). In addition, there was a signal in the 1060 cm^-1^ region that was characteristic of the C-O stretching vibration of the pyranose ring present in glucose (*7*). Absorption in the 880 cm^-1^ region indicates the β-type configuration of this polysaccharide (*7*, *29*), which was weakened in the sulfonated derivatives.

In the spectra of the sulfonated derivatives (LAS-S), bands characteristic of sulfur and sulfonyl hydroxide groups appear unlike bands observed in the LAS-N spectrum. In Fig. 2, it is possible to verify that the characteristic O-H stretching band in the region between 4000 and 2500 cm^-1^ decreases in intensity following the sulfonation reaction (*16*). The reduction in the intensity of this band can be attributed to the differences in absorption in the infrared region between the hydroxyl constituents of lasiodiplodan and the hydroxyls present in the sulfonyl hydroxide group (S(=O)_2_-(OH)).

After sulfonation, the characteristic bands of C-H and O-H deformation vibrations at 1400  cm^-1^ in the LAS-N sample disappear (*29*), which indicates the occurrence of a structural change in the molecule caused by derivatization. Another aspect observed is the appearance of a band at 1250 cm^-1^ in the sulfonated samples, which is attributed to the asymmetric (strong) stretching vibration S=O, associated with the distribution of sulfonyl hydroxide groups in the molecule (*30*). Fig. 2 also shows an increase in band intensity at 1250 cm^-1^ concomitant with an increase in the sample’s DS.

At 810 cm^-1^, a band corresponding to the symmetrical vibration C-O-S, associated with the C-O-SO_3_ group is verified, which confirms sulfonation (*7*, *25*). A higher intensity of this band is found in the lasiodiplodan samples with higher DS, as shown in Fig. 2.

### Scanning electron micrographs of LAS-N and LAS-S

**Fig. 3:**
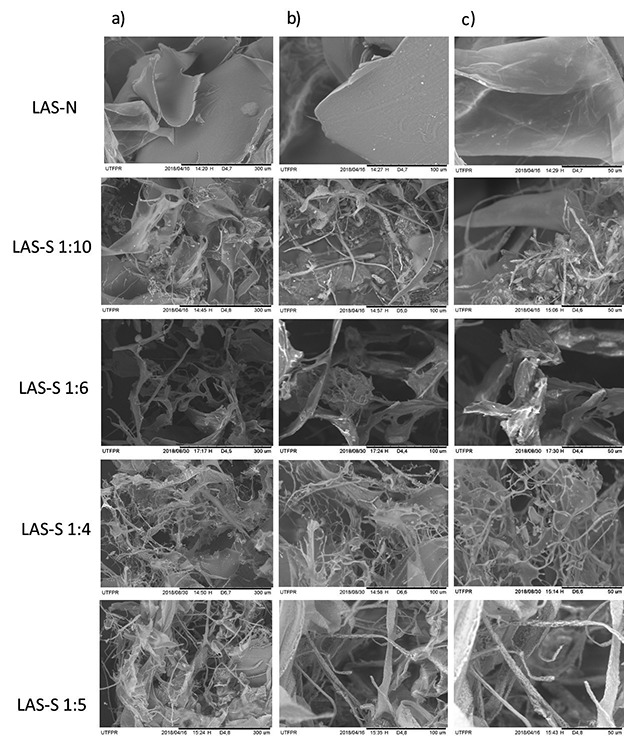
Scanning electron microscopy (SEM) micrographs of native lasiodiplodan (LAS-N) and sulfonated lasiodiplodan (LAS-S) with different chlorosulfonic acid/pyridine ratio: 1:6 (DS=1.01), 1:4 (DS=1.42) and 1:5 (DS=1.61) at magnifications of: a) 300×, b) 800× and c) 1200×. DS=degree of substitution

The SEM micrographs of LAS-N and the sulfonated derivative samples are shown in Fig. 3 at 300×, 800× and 1200× magnification. SEM shows that LAS-N has a nonuniform surface containing irregular folds and edges (Fig. 3a and Fig. 3b, LAS-N) with portions similar to thin and translucent films with torsions along the extension (Fig. 3c, LAS-N). Sulfonation promoted morphological changes on the lasiodiplodan surface, leading to the appearance of heterogeneous fibrillary structures along the surface area. The DS of the sulfonated samples appears to have influenced the surface morphology of lasiodiplodan, as observed in the samples with the highest DS (LAS-S 1:4 with DS=1.42 and LAS-S 1:5 with DS=1.61), with greater number of fibrillary structures associated with thin and translucent films. On the other hand, samples with lower DS (LAS-S 1:10 with DS=0.15 and LAS-S 1:6 with DS=1.02) had a smaller number of fibrillary structures along the surface area. The morphological differences among the LAS-S samples with higher DS than those with lower DS may be associated with the intensity of the sulfonation reaction. To obtain LAS-S derivatives with higher degrees of sulfonation, it was necessary to use higher concentrations of chlorosulfonic acid in preparing the sulfonating reagent.

### Thermal profile of lasiodiplodan samples

**Fig. 4:**
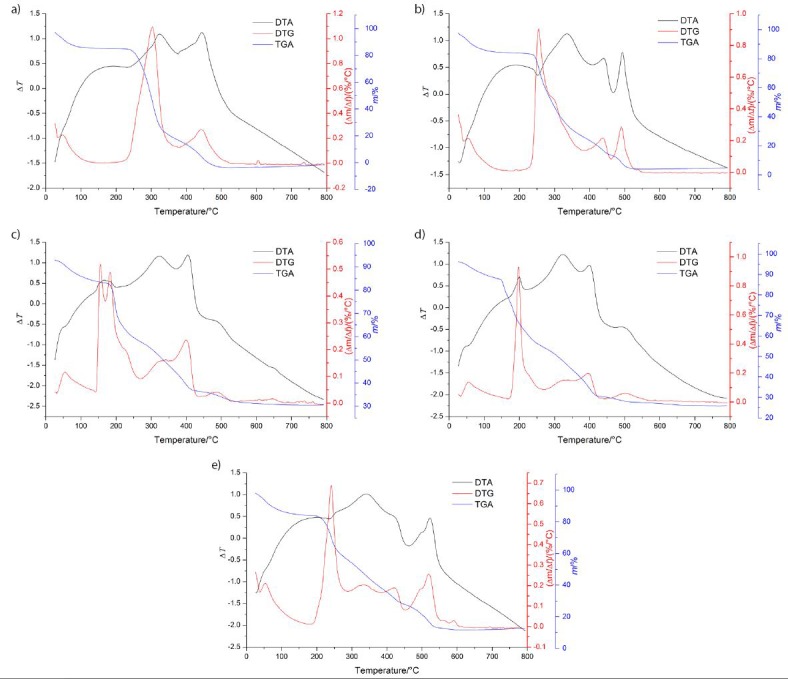
Derivative thermogravimetry (DTG), differential thermal analysis (DTA) and thermogravimetric analysis (TGA) profiles of: a) native lasiodiplodan (LAS-N), and b) sulfonated lasiodiplodan (LAS-S) samples with different chlorosulfonic acid/pyridine ratio: b) 1:10 (DS=0.15), c) 1:6 (DS=1.02), d) 1:4 (DS=1.42) and e) 1:5 (DS=1.61). DS=degree of substitution

The thermal profiles (DTG, DTA and TGA) of LAS-N and LAS-S are shown in Fig. 4. LAS-N was stable up to 200 °C and showed three stages of mass loss as can be verified in DTG, TGA and DTA curves (Fig. 4). The first mass loss (12%) occurred up to 122 °C, corresponding to the elimination of water by hydration. The second stage of mass loss (83%) occurred between 200 and 377 °C (TGA curve), and was indicated by an exothermic peak at 301 °C (DTA curve) and corresponded to the thermal decomposition of the sample. The third stage of mass loss (100%) occurred in the temperature range 376 to 548 °C (TGA curve) with an exothermic peak at 442 °C (DTA curve), and is attributable to the final decomposition (carbonization) of the sample.

Sulfonation promoted changes in the thermal profile of lasiodiplodan. The LAS-S samples showed four stages of mass loss, while the LAS-N had three. The first stage of mass loss (14%), typical of intracellular water elimination, occurred up to 160 °C, with this range being higher than the water elimination temperature range of the non-sulfonated sample (up to 122 °C). This phenomenon can be attributed to the reduction of the macromolecule hydrophobicity after introduction of the sulfonyl hydroxide group (S(=O)_2_-OH) by sulfonation. In fact, this behaviour corroborates the increase in water solubility of the sulfonated derivatives in relation to LAS-N.

The second stage occurred between 160 and 290 °C, with mass losses ranging from 44% (LAS-S 1:10 with DS=0.15) to 43% (LAS-S 1:5 with DS=1.61), as evidenced by an exothermic peak (DTA curve) at 200 °C, and attributed to the thermal decomposition of the sample (Fig. 4). The third stage of mass loss (73%) occurred between 290 and 460 °C, and it corresponds to the oxidative decomposition of the sample, as evidenced by the exothermic peak (DTA curve) at 400 °C. The final stage, corresponding to carbonization, occurred between 440 and 570 °C, as revealed by the exothermic peak at 500 °C (DTA curve).

In general, sulfonation of lasiodiplodan contributed to a small increase in thermal stability. This behaviour is evidenced by the increase in the temperature range corresponding to the oxidation of the biopolymer (from 377 to 460 °C), as well as an increase in the final carbonization temperature (548 to 570 °C).

### Antimicrobial activity of LAS-N and LAS-S

According to Table 2, it is possible to verify that both LAS-N and LAS-S samples showed potential inhibition against both the investigated yeasts and bacteria. LAS-N had a fungistatic effect against *C. albicans* and *C. tropicalis* at concentrations of 0.15 and 0.26 mg/mL, respectively. LAS-N also showed bacteriostatic effect against *L. monocytogenes* (Gram-positive) at a minimum inhibitory concentration (MIC) of 0.15 mg/mL, and a bactericidal effect against *E. coli* (Gram-negative) with minimum bactericidal concentration (MBC)=0.05 mg/mL. The sulfonated samples also showed antimicrobial activity against all of the studied microorganisms, with the exception of *S. aureus*, which showed resistance against all the tested samples (Table 2). The degree of substitution seems to influence the antimicrobial potential, however, there was no linear correlation between the DS and inhibitory capacity. In this context, the LAS-S sample with DS=1.42 showed no inhibitory activity against the evaluated microorganisms, whereas the sample with a lower DS (0.15) showed fungistatic activity (MIC=0.26 mg/mL) against *C. albicans* and *C. tropicalis*. Likewise, LAS-S samples with DS=1.02 (MIC=0.20 mg/mL) and 1.61 (MIC=0.26 mg/mL) had fungicidal activity against *C. albicans*, but *C. tropicalis* was not inhibited by the sample with higher DS (1.61). Sulfonation does not appear to be effective against Gram-positive bacteria (*S. aureus* and *L. monocytogenes*). Only *L. monocytogenes* was inhibited by the LAS-S sample with a DS=1.02 (MIC=0.26 mg/mL). Among all the studied sulfonated samples, the sample with DS=1.02 showed the highest antimicrobial potential. It is important to highlight that sulfonation producing LAS-S of DS=1.02 and 1.61 promoted antimicrobial activity against *S. enterica* Typhimurium (Gram-negative), an effect not observed by the native molecule (LAS-N). Another aspect to be considered is that Gram-negative bacteria were more resistant than Gram-positive bacteria due to their cell wall chemistries (*31*).

**Table 2:** Antimicrobial potential of native (LAS-N) and sulfonated lasiodiplodan (LAS-S) samples with different degrees of substitution (DS) against different strains of bacteria and yeasts

Microorganism	Sample				
LAS-N	LAS-S^1:10^	LAS-S^1:6^	LAS-S^1:4^	LAS-S^1:5^
		DS		
0.0	0.15	1.02	1.42	1.62
*Candida albicans*	# (0.15)	# (0.26)	++ (0.20)	-	++ (0.26)
*Candida tropicalis*	# (0.26)	# (0.26)	# (0.20)	-	-
*Staphylococcus aureus*	-	-	-	-	-
*Listeria monocytogenes*	# (0.15)	-	# (0.26)	-	-
*Salmonella enterica* Typhimurium	-	-	# (0.26)	-	# (0.26)
*Escherichia coli*	++ (0.05)	-	# (0.20)	-	# (0.10)

Superscripts in LAS-S denote different ratios of chlorosulfonic acid/pyridine (1:10, 1:6, 1:4, 1:5) used for sulfonation. - resistance, ++ bactericidal or fungicidal (cell death promotion), # bacteriostatic or fungistatic (inhibition of cell growth). Values in brackets correspond to the tested sample concentrations (mg/mL)

### Antioxidant potential of LAS-N and LAS-S

**Fig. 5:**
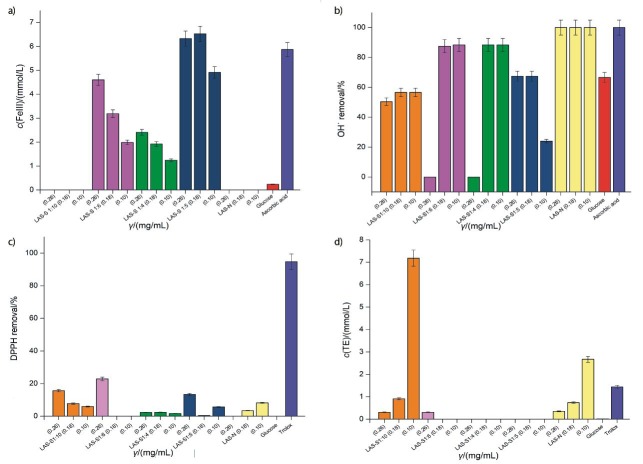
Antioxidant activity of native (LAS-N) and sulfonated lasiodiplodan (LAS-S) with different degrees of substitution (DS) and chlorosulfonic acid/pyridine ratio: 1:5 (DS=1.61), 1:4 (DS=1.42), 1:6 (DS=1.02) and 1:10 (DS=0.15) determined by: a) ferric reducing antioxidant ability (FRAP), b) OH˙, c) DPPH and d) ABTS radical scavenging potential. TE=Trolox equivalent

The results obtained for FRAP antioxidant potential are shown in Fig. 5a. Glucose showed very low FRAP potential (0.24 mg/mL), while LAS-N at the evaluated concentrations showed no FRAP capacity at all. These results indicate that there is different antioxidant activity behaviour between the glucose in its monosaccharide form and the glucose as a constituent of the β-glucan. Ascorbic acid showed a high FRAP potential (5.87 mg/mL), which was to be expected as it is used universally as a reference antioxidant standard.

Sulfonation promoted ferric reducing antioxidant ability, except for the sample with a low DS (0.15), which did not have FRAP potential at all. The introduction of a larger number of sulfonyl hydroxide groups in the macromolecule contributed to a higher FRAP potential. LAS-S samples with the highest DS (1.42 and 1.61) had the highest FRAP potential, 6.53 mg/mL (*γ*=0.18 mg/mL) and 4.6 mg/mL (*γ*=0.26 mg/mL), respectively.

Another aspect to be highlighted is a correlation between the concentration of the sulfonated polysaccharide and the FRAP activity in the samples with different DS values. A dose-dependent relationship between sulfonated polysaccharide concentrations and ferric reducing power has also been reported by Li and Shah (*23*), who evaluated the FRAP potential of sulfonated derivatives of a polysaccharide extracted from *Pleurotus enryngii*, and an exopolysaccharide obtained from *Streptococcus thermophilus* ASCC 1275.

The OH˙ scavenging capacity of all studied samples is shown in Fig. 5b. Among the tested antioxidant evaluation methods, LAS-N and LAS-S samples showed higher antioxidant capacity when evaluated by the OH˙ radical scavenging potential. As shown in Fig. 5b, ascorbic acid removed 100% of the OH˙ radicals. Glucose also had appreciable OH˙ scavenging activity (66.66%). Likewise, LAS-N was able to eliminate 100% OH˙ radicals at all of the concentrations tested (0.26, 0.18 and 0.10 mg/mL), which indicates that LAS-N was a good candidate for scavenging OH˙ radicals.

LAS-S samples also showed high OH˙ scavenging activities, at concentration 0.10 mg/mL and DS=0.15, 1.02 and 1.42 the OH˙ removal was 56.59, 88.37 and 88.00%, respectively, while at concentration of 0.18 mg/mL and DS=1.61 it was 67.44%. These results were different to those observed with FRAP method. Sulfonation did not potentiate OH˙ removal capacity as high as the native sample (LAS-N).

Unlike the behaviour observed with the FRAP potential, a direct correlation between the OH˙ scavenging capacity and DS was not observed. Likewise, the sulfonated polysaccharide dose did not influence the OH˙ scavenging capacity, since the higher concentrations tested did not increase this activity.

The DPPH radical scavenging capacity of LAS-N, LAS-S, glucose and Trolox (standard) samples is shown in Fig. 5c. Trolox standard was able to remove almost 100% DPPH radical, while glucose did not demonstrate any activity. Both LAS-N and LAS-S samples showed poor antioxidant activity by the DPPH method, and there was no dose-dependent relationship between the DS and β-glucan concentration. LAS-N presented maximum removal capacity (8.21%) at a concentration of 0.10 mg/mL. The highest values of DPPH removal were found in LAS-S samples with lower DS values (DS=0.15 and 1.02), removing 15.67 and 22.88%, respectively, and 0.26 mg/mL for LAS-S with DS=1.61.

The ABTS radical scavenging capacity of LAS-N, LAS-S, glucose and Trolox samples is shown in Fig. 5d. LAS-N and the sulfonated samples with low DS (0.15) had antioxidant potential against the ABTS radical. The highest scavenging activity of the ABTS radical was found in the assays with lower concentrations (0.10 mg/mL) of LAS-N (2.68 mmol/L) and LAS-S (7.18 mmol/L). An increase in the concentration of polysaccharide did not promote greater antioxidant capacity. Sulfonated lasiodiplodan with lower DS (0.15) showed a higher ABTS radical scavenging capacity (168%) than LAS-N.

The antioxidant activities of LAS-N and LAS-S evaluated by the DPPH and ABTS radical scavenging capacity were inferior to those found by the FRAP and OH˙ removal procedures. Such behaviour may possibly be attributable to the use of ethanol as solvent in the DPPH and ABTS radical antioxidant assay protocols, as polysaccharides can be insolubilized in alcohols.

### Cytotoxicity on Saccharomyces cerevisiae strains

As shown in Table 3, the reference standard, menadione (1,4-naphthoquinone or vitamin K3), demonstrated lethal cytotoxicity against *S. cerevisiae* strains, even at very low concentrations. In fact, menadione is an important agent that induces oxidative stress, which contributes to the increase of the production of reactive oxygen species (ROS), and causes ROS levels to exceed the endogenous antioxidant capacity (*21*, *32*). On the other hand, LAS-N and the sulfonated samples did not demonstrate lethal cytotoxicity against the *S. cerevisiae* strains at the evaluated concentrations. Only LAS-N at 0.20 mg/mL showed an inhibitory effect (biostatic) for 24 h, both against the wild and mutant strains. Similarly, sulfonated samples with the lowest DS (0.15 and 1.02) did not have biocidal activity, inhibiting only cellular activity for 24 h at concentrations of 0.20 and 0.26 mg/mL.

**Table 3:** Oxidative stress by native (LAS-N) and sulfonated lasiodiplodan (LAS-S) with different degrees of substitution (DS) towards various strains of *Saccharomyces cerevisiae*

*S. cerevisiae* strain	LAS-N	LAS-S				*c*(menadione/(mmol/L)
0.0	0.15	DS 1.02	1.42	1.61
	
Standard	# (0.20)	# (0.26)	# (0.20)	-	-	++ (18.5)
YLL060C	# (0.20)	# (0.26)	# (0.20)	-	# (0.26)	++ (18.5)
YIR038C	# (0.20)	# (0.20)	# (0.20)	-	-	++ (18.5)
YSL101C	# (0.20)	# (0.20)	# (0.20)	-	# (0.26)	++ (18.5)

Standard=*Saccharomyces cerevisiae* ex r.f. *bayanus*. - resistance, ++ biocidal activity, # biostatic activity. Values in brackets correspond to the tested sample concentrations (mg/mL)

The sulfonate derivatives with the highest DS (1.42 and 1.61) showed lower capacity for induction of oxidative stress. In this case, the sulfonated sample with DS=1.42 did not inhibit any of the evaluated strains. LAS-S with the highest DS (1.61) promoted biostatic activity at concentration 0.26 mg/mL only on mutant strains YLL060C and YSL101C. Although LAS-N and LAS-S demonstrated antioxidant activity, especially hydroxyl radical scavenging ability, and ferric ion reducing capacity, there was probably no complete inhibition of the production and accumulation of certain ROS by the mutant cells of *S. cerevisiae* induced by lasiodiplodan. It is important to note that even though there is no elimination of an imbalance between ROS accumulation and antioxidant activity, there was no lethal effect on the mutant strains, which are unable to produce glutathione (*20*), suggesting a low toxicity of lasiodiplodan in relation to induction of oxidative stress.

## CONCLUSIONS

Four sulfonated derivatives were obtained by sulfonation of the native exopolysaccharide lasiodiplodan (LAS-N). The degree of substitution of the derivatives ranged from 0.15 to 1.61, and sulfonation was confirmed by FTIR with bands at 1250 (strong asymmetrical elongation vibration S=O) and 810 cm^-1^ (symmetrical vibration C-O-S, associated with the group C-O-SO_3_). The introduction of O-SO_3_H groups into the macromolecule contributed to the increase in the solubility of lasiodiplodan, concomitantly with the increase of the degree of sulfonation of the sample. Sulfonation appears to be a prominent mechanism for the functionalization of lasiodiplodan, including potentiation of antioxidant and antimicrobial activities. It increased the antimicrobial activity, especially against *Candida albicans* and *Salmonella enterica* Typhimurium, exerting a fungicidal and bacteriostatic effect, respectively. Sulfonated derivatives showed high FRAP potential, and both native and sulfonated samples did not demonstrate lethal cytotoxicity against the wild and mutant strains of *Saccharomyces cerevisiae*. Sulfonated lasiodiplodan samples showed the highest thermal stability compared to the native non-chemically modified polysaccharide. In addition, derivatization by sulfonation promoted morphological changes on the surface structure of the biopolymer, including the appearance of heterogeneous fibrillary structures along the surface area.
